# Perceptions of the Harm of Cigarettes, Mental Health, and Quality of Life Among Transgender Adults Who Smoke Menthol Cigarettes: Results from Wave 5 of the Population Assessment for Tobacco and Health (PATH) Study

**DOI:** 10.3390/ijerph21121618

**Published:** 2024-12-03

**Authors:** Nichelle Brown, Heesung Shin, Sabrina L. Smiley

**Affiliations:** Division of Health Promotion and Behavioral Science, School of Public Health, College of Health and Human Services, San Diego State University, 5500 Campanile Dr., San Diego, CA 92182, USA

**Keywords:** transgender, tobacco, cigarette smoking, menthol, harm perceptions, PATH

## Abstract

Sexual and gender minority (SGM) individuals in the United States are at greater risk for combustible tobacco use and mental health problems compared to heterosexual and cisgender individuals. National data comparing associations of menthol cigarette use and mental health among transgender and cisgender individuals in the United States are lacking. The goals of the current study were to (1) characterize transgender and cisgender individuals who smoke menthol cigarettes, and (2) investigate cross-sectional associations between gender identity, harm perceptions of cigarettes, mental health experiences, and quality of life. Data were drawn from Wave 5 of the Population Assessment of Tobacco and Health (PATH) Study (2018–2019). A total of 3989 cisgender (mean [SD] age, 40.47 [0.32] years) and transgender (mean [SD] age, 38.36 [0.09] years) participants who reported current (past 30-day) menthol cigarette use were included in the analysis. Comparing transgender to cisgender groups, significant differences were found in associations between harm perceptions of cigarettes (adjusted odds ratio [AOR] 0.07; 95% CI: 0.02–0.28) and reported depression (AOR 4.57; 95% CI: 1.36–15.33). Collectively, these findings provide evidence that transgender adults who smoke menthol cigarettes are less likely than cisgender adults who smoke menthol cigarettes to perceive smoking as harmful to health and more likely to report recent experiences of depression.

## 1. Introduction

Menthol is characterized by its ability to mask the harshness of nicotine and increase the bioavailability of nicotine. [1] Research has shown that menthol cigarette use is associated with increased nicotine dependence. [1,2] Menthol cigarettes are a major source of morbidity and mortality in the United States [2,3,4,5]. Although cigarette use has steadily declined over several decades, [6,7] the decline has been greater among nonmenthol cigarettes (52.9% from 2000 to 2018) than for menthol cigarettes (26.1% from 2000 to 2018) [8]. In 2022, 37.52% of U.S. adults who smoked cigarettes in the past month used menthol [9].

Menthol cigarette use disproportionately affects priority populations, including Black Americans, young adults (18–34), sexual and gender minority adults (SGM), and individuals with mental health problems [2,10,11]. SGM adults, specifically, have a higher estimated prevalence of menthol cigarette use compared to their heterosexual cisgender counterparts [10]. Studies often group transgender and gender-diverse people with sexual minorities, even though they do not have the same experiences. There is a lack of research investigating menthol cigarette use among transgender and gender-diverse people.

Low harm perceptions of cigarettes has been shown to be associated with current, past 30-day, cigarette use [12]. Targeted marketing and Exposure to misinformation via social media may play a role in influencing harm perceptions in this community [10,13]. Research has also shown that experiences of minority stress (i.e., stigma, discrimination, and identity concealment) among transgender and gender-diverse individuals can override harmful perceptions of smoking, leading to continued use [14]. Few studies have examined the harmful perceptions of menthol cigarettes, especially among transgender and gender-diverse people [12].

Mental health conditions, such as anxiety and depression, have been shown to be associated with tobacco use [15,16,17,18]. Specifically, those with mental health conditions have been shown to have increased menthol cigarette use [11]. Transgender and gender-diverse people report experiences of anxiety and depression at higher rates than cisgender people [19]. Anxiety and depression contribute to low quality of life, which has been shown to be associated with increased cigarette use [20]. A study found that transgender men reported a lower quality of life compared to cisgender men and women [21]. Studies investigating the quality of life among transgender people who smoke cigarettes, specifically menthol cigarettes, are limited.

National data comparing associations of menthol cigarette use and mental health among transgender and cisgender individuals in the United States are lacking. The goals of the current study were to (1) characterize transgender and cisgender individuals who smoke menthol cigarettes, and (2) investigate harm perceptions of cigarettes, mental health experiences, and quality of life of people who smoke menthol cigarettes, by gender identity.

## 2. Materials and Methods

### 2.1. Data Source

Study data employed Wave 5 of the adult surveys (≥18 years) available in the restricted-use files of the Population Assessment of Tobacco and Health (PATH) Study. A nationally representative longitudinal cohort study of adults (≥18 years) and youth (12–17 years) in the United States, the PATH Study uses audio computer-assisted self-interviews (English and Spanish) to collect self-reported data information on commercial tobacco use patterns and associated health behaviors [22]. Further details regarding the PATH Study design and methodology are published elsewhere [22]. Wave 5 data collection (December 2018 to November 2019) resulted in a nationally representative sample of 34,309 adults (≥18 years; 88.0% weighted response rate). The PATH Study was conducted by Westat and approved by the Westat Institutional Review Board. All respondents ages ≥18 years provided informed consent.

### 2.2. Measures

Menthol Cigarette Use—Participants reported whether any of the cigarettes they smoked in the past 30 days were mint or menthol flavored. Consistent with prior work [23], participants who responded “yes” were considered menthol smokers, whereas participants who responded “no” were considered non-menthol smokers.

Sociodemographic Characteristics—Participants reported a variety of sociodemographic characteristics, including age, gender identity, race, ethnicity, sexual identity (“straight”, “lesbian or gay”, “bisexual”, or “something else”), and annual household income. Transgender identity was assessed through the prompt: “Some people describe themselves as transgender when they experience a different gender identity from their sex at birth. For example, a person born into a male body, but who feels female or lives as a woman would be transgender. Do you consider yourself to be transgender?” All respondents who responded “yes” to this question were considered to be transgender.

Perceived Harm of Cigarette Use—Perceptions of harm of cigarette smoking were assessed with the question, “How harmful do you think cigarettes are to health?”, on a 5-point Likert scale ranging from 1 (not at all harmful) to 5 (extremely harmful). For these analyses, perceptions of the harm of cigarette smoking were dichotomized into “Not at all harmful” and “Harmful”.

Perceived Mental Health—PATH uses items from the Global Appraisal of Individual Needs (GAIN) internalizing mental health subscale (alpha = 0.96) to measure anxiety and depression. [24] Participants were asked, “When was the last time that you had significant problems with feeling very anxious, nervous, tense, scared, panicked, or like something bad was going to happen?” and “When was the last time that you had significant problems with feeling very trapped, lonely, sad, blue, depressed, or hopeless about the future?”. Responses to these questions included, “Past month”, ”2 to 12 months ago”, “Over a year ago”, and “Never”. For multivariate logistic regression, responses were dichotomized into “Never and over a year ago” and “Past month to 12 months year ago”.

Quality of Life—Participants were asked to rate their QoL on a 5-point Likert scale from 1 (excellent) to 5 (poor), with higher scores indicating worse QoL. For multivariate logistic regression, responses were dichotomized into “Poor to Good” and “Very Good or Excellent”.

### 2.3. Data Analysis

Differences between transgender and cisgender respondents on all variables were assessed using bivariate analyses. Chi-square tests and multivariate logistic regression models were used to determine associations between gender identity and significant variables of interest. For each item, responses of ‘don’t know’ or ‘refused’ were treated as missing data. The PATH Study population weights were used to adjust for the complex study design, including oversampling, attrition, and non-response so that estimates are representative of the U.S. civilian, noninstitutionalized population [22]. Estimates were calculated with balanced repeated replication methods using Fay’s adjustment value of 0.3 [25,26]. All analyses used listwise deletion to address missing data and were conducted using SAS 9.4 (SAS Institute Inc., Cary, NC, USA).

## 3. Results

### 3.1. Sample Demographics

As shown in Table 1, the current study’s total sample comprised 3989 cisgender (n = 3950) and transgender (n = 39) adults who smoke menthol cigarettes, and the mean age was 40.5 (SE = 0.31). The sample was predominantly non-Hispanic/Latine White (63.63%), identified as “straight” (87.48%), and had an annual household income of less than USD 50,000 (72.84%). Sexual identity and annual household income differed significantly by gender identity. A higher percentage of cisgender adults who smoke menthol cigarettes identified as “straight” (87.69% versus 67.13%; *p* < 0.01) compared to transgender adults who smoke menthol cigarettes. A higher percentage of transgender adults who smoke menthol cigarettes reported an annual household income of less than USD 50,000 (90.69% versus 72.66%; *p* = 0.02), compared to cisgender adults who smoke menthol cigarettes.

### 3.2. Cigarette Smoking and Harm Perceptions

There were 34,309 adult (aged 18 years and above) respondents who provided data at Wave 5. A total of (28.41%; n = 9748) adults who smoke cigarettes identified as cisgender (n = 9657) or transgender (n = 91). The study sample included 3989 cisgender (n = 3950) and transgender (n = 39) adults who smoke menthol cigarettes. Harm perceptions of cigarettes are shown in Table 2. Transgender respondents were significantly less likely than cisgender respondents to perceive cigarettes as harmful to their health (77.44% versus 98.13%; *p* < 0.01).

### 3.3. Mental Health and Quality of Life

Self-reported mental health and quality of life are shown in Table 3. Transgender respondents were significantly more likely than cisgender respondents to report feeling anxious (56.26% versus 26.25%; *p* < 0.01) and depressed (54.26% versus 23.38%; *p* < 0.01) in the past month. Compared to cisgender respondents, transgender respondents were more likely to rate their quality of life as poor (10.97% versus 1.71%; *p* < 0.01).

### 3.4. Multivariable Models

After adjusting for sociodemographic characteristics (Table 4), transgender respondents had lower odds of perceiving cigarettes as harmful to their health (adjusted odds ratio [AOR] 0.07; 95% CI: 0.02–0.28), and higher odds of reporting recent experiences of depression (AOR 4.57; 95% CI: 1.36–15.33). Black respondents had lower odds of perceiving cigarettes as harmful to their health (AOR 0.43; 95% CI: 0.24–0.74), and lower odds of reporting recent experiences of anxiety (AOR 0.52; 95% CI: 0.43–0.63) and depression (AOR 0.57; 95% CI: 0.45–0.72). Bisexual respondents had higher odds of reporting recent experiences of anxiety (AOR 2.54; 95% CI: 1.85–3.49) and depression (AOR 2.35; 95% CI: 1.66–3.32). There were no significant differences in quality of life by gender identity.

## 4. Discussion

This study used a nationally representative sample of adult surveys from Wave 5 of the PATH Study (2018–2019) to (1) characterize transgender and cisgender individuals who smoke menthol cigarettes, and (2) investigate harm perceptions of cigarettes, mental health experiences, and quality of life of people who smoke menthol cigarettes, by gender identity. A better understanding of the relationship between gender identity, harmful perceptions of cigarettes, mental health, quality of life, and menthol cigarette use is important, given menthol cigarettes are a major source of morbidity and mortality in the United States [2,3,4,5].

### 4.1. Harm Perceptions of Cigarettes

Few studies have assessed the perceived harm of cigarettes among transgender people. A previous study found that transgender people who smoke cigarettes perceive smoking as harmful, but smoke anyway due to minority stress experiences [14]. Extending prior research [14], this study found that transgender people who smoke menthol cigarettes were less likely to perceive smoking as harmful to their health compared to their cisgender counterparts. Research among cigarette smokers has also shown that menthol cigarette use is higher among Black adults compared to White adults [27]. In this study of menthol cigarette smokers, Black respondents were less likely to perceive smoking as harmful to health compared to White respondents. More robust and longitudinal studies are needed to determine the relationship between gender identity, harm perceptions of cigarettes, mental health, and quality of life. These studies are especially needed in Black and Brown gender-diverse populations.

### 4.2. Mental Health

Adults identifying as transgender who smoke menthol cigarettes reported significantly higher odds of depression in the past year relative to their cisgender counterparts. Mental health problems account for a significant proportion of the disease burden among transgender people and are strongly correlated with substance use [28]. This is similar to other research findings [29] that link poor mental health to cigarette use among SGM. Future studies should use the Gender Minority Stress Framework [30] to investigate specific mental health stressors (i.e., stigma-related stress) of transgender adults with respect to menthol cigarette smoking as a coping mechanism.

Black people who smoke menthol cigarettes were less likely to report recent experiences of anxiety and depression. These findings align with the “black-white mental health paradox”, which posits that, despite experiencing more stress, Black people report lower or similar rates of psychiatric disorders compared to White people [31,32]. Future tobacco studies, among transgender adults, investigating mental health should account for exposure to stressors and highlight resilience. This is especially important for Black transgender people since the Gender Minority Stress Framework and “black-white mental health paradox” both include elements of resilience that are protective against stressors [29,31].

Compared to heterosexual people, menthol cigarette use is higher among sexual minority people [10]. Studies of mental health among sexual minority people have found that, in addition to having higher rates of anxiety and depression compared to heterosexual people, bisexual people report higher or similar rates of anxiety and depression compared to lesbian/gay and heterosexual people [33]. This study of menthol cigarette smokers had similar findings regarding bisexual people reporting anxiety and depression. These findings further emphasize that tobacco studies of sexual minority people should take identity into account, rather than aggregating experiences into one group.

### 4.3. Limitations

Several important limitations should be noted. First, study data were obtained via self-report, and future research should include biochemical verification of nicotine use. Second, menthol cigarette use was assessed via self-report past 30-day use of menthol/mint cigarettes, with no indication of frequency or intensity. Third, the small sample size of transgender people who smoke menthol cigarettes limits its generalizability in the population of gender-diverse people who smoke menthol cigarettes. Fourth, due to the small sample size of transgender people and confidentiality, we could not stratify gender identity by race and ethnicity for subgroup analyses.

## 5. Conclusions

This national study characterized transgender and cisgender people who smoke menthol cigarettes. In adjusted analyses, transgender adults who smoke menthol cigarettes were less likely than their cisgender counterparts to perceive smoking as harmful to health and more likely to report recent experiences of sadness or depression. These results suggest that menthol cigarette cessation and prevention programs should include evidence-based therapeutic aspects to address identity-related mental health experiences. Study results should also serve as a call to action to include and increase the representation of transgender people in nicotine and commercial tobacco policy and research. Such research can inform the development of public health interventions to prevent menthol cigarette use and support cessation among transgender people who smoke in the U.S.

## Figures and Tables

**Table 1:** Demographic characteristics of the study sample.

Characteristic	Total		Transgender		Cisgender		*p*-Value
	(n = 3989)		(n = 39)		(n = 3950)		
	Weighted %	n	Weighted %	n	Weighted %	n	
Sexual Identity							<0.01
Straight	87.48	3375	67.13	19	87.69	3356	
Lesbian/Gay	3.88	154	3.74	4	3.88	150	
Bisexual	7.08	365	12.60	7	7.02	358	
Something else	1.55	72	16.54	9	1.40	63	
Missing	--	23	--	--	--	23	
Race							0.91
White	63.63	2371	68.28	23	63.59	2348	
Black	26.97	1085	23.73	7	27.01	1078	
Asian/Other/Multiracial	9.39	433	7.99	7	9.41	426	
Missing	--	100	--	2	--	98	
Latine							0.18
Yes	17.75	763	10.79	9	17.83	754	
No	82.25	3169	89.21	30	82.17	3139	
Missing	--	57	--	--	--	57	
Age							0.40
Mean (SE)	40.45 (0.31)		38.36 (0.09)		40.47 (0.32)		
Income							0.02
Low: Less than 50,000	72.84	2895	90.69	32	72.66	2863	
Middle/High: 50,000 or more	27.16	915	9.31	5	27.34	910	
Missing	--	179	--	2	--	177	

**Table 2:** Harm perceptions of cigarettes.

Characteristic	Total		Transgender		Cisgender		*p*-Value
	(n = 3989)		(n = 39)		(n = 3950)		
	Weighted %	n	Weighted %	n	Weighted %	n	
Cigarettes harm to health							<0.01
Not at all harmful	2.08	94	22.56	7	1.87	87	
Harmful	97.92	3882	77.44	32	98.13	3850	
Missing	--	13	--	--	--	13	

**Table 3:** Self-reported mental health and quality of life.

Characteristic	Total		Transgender		Cisgender		*p*-Value
	(n = 3989)		(n = 39)		(n = 3950)		
	Weighted %	n	Weighted %	n	Weighted %	n	
Last time feeling very anxious, nervous, tense, scared, panicked or like something bad was going to happen							<0.01
Past month	26.55	1157	56.26	18	26.25	1139	
2–12 months ago	18.36	726	10.44	7	18.44	719	
Over a year ago	13.37	527	18.32	9	13.32	518	
Never	41.71	1556	14.98	5	41.99	1551	
Missing	--	23	--	--	--	23	
Last time feeling very trapped, lonely, sad, blue, depressed or hopeless about the future							<0.01
Past month	23.70	1026	54.26	17	23.38	1009	
2–12 months ago	17.82	719	24.49	13	17.76	706	
Over a year ago	17.84	703	10.29	5	17.92	698	
Never	40.64	1520	10.96	4	40.94	1516	
Missing	--	21	--	--	--	21	
Quality of life							<0.01
Excellent	11.98	521	31.65	7	11.78	514	
Very good	31.55	1221	14.83	9	31.72	1212	
Good	33.71	1520	31.65	10	38.77	1510	
Fair	15.96	642	10.89	7	16.02	635	
Poor	1.80	75	10.97	6	1.71	69	
Missing	--	10	--	--	--	10	

**Table 4:** Multivariable logistic regression models.

Variables	Model 1: Harm Perception		Model 2: Reported Anxiousness		Model 3: Reported Sadness/Depression		Model 4: Quality of Life	
	OR	95% CI	OR	95% CI	OR	95% CI	OR	95% CI
Gender Identity								
Transgender	0.07 *	(0.02–0.28)	2.48	(0.99–6.22)	5.30 *	(1.95–14.39)	1.30	(0.57–2.97)
Cisgender	1.00		1.00		1.00		1.00	
Adjusted Odds Ratios	AOR	95% CI	AOR	95% CI	AOR	95% CI	AOR	95% CI
Gender Identity								
Transgender	0.07 *	(0.02–0.32)	2.17	(0.65–7.25)	4.57 *	(1.36–15.33)	1.14	(0.49–2.67)
Cisgender	1.00		1.00		1.00		1.00	
Sexual Identity								
Straight	1.00		1.00		1.00		1.00	
Lesbian/Gay	1.14	(0.28–4.67)	1.56	(0.97–2.51)	2.62 *	(1.54–4.44)	1.23	(0.68–2.24)
Bisexual	2.32	(0.69–7.78)	2.54 *	(1.85–3.49)	2.35 *	(1.66–3.32)	1.05	(0.73–1.50)
Something else	0.85	(0.23–3.15)	1.43	(0.74–2.73)	1.39	(0.68–2.86)	0.64	(0.31–1.32)
Race								
White	1.00		1.00		1.00		1.00	
Black	0.43 *	(0.24–0.74)	0.52 *	(0.43–0.63)	0.57 *	(0.45–0.72)	0.96	(0.74–1.24)
Asian/Other/Multiracial	0.93	(0.43–2.02)	0.87	(0.63–1.20)	0.94	(0.67–1.32)	1.53 *	(1.09–2.13)
Latine								
Yes	0.94	(0.44–1.99)	0.84	(0.66–1.05)	0.91	(0.70–1.17)	1.07	(0.77–1.48)
No	1.00		1.00		1.00		1.00	
Age								
Units = 1 Year	1.05 *	(1.02–1.07)	0.98 *	(0.97–0.98)	0.99 *	(0.98–0.99)	1.01 *	(1.00–1.02)
Income								
Low: Less than 50,000	1.00		1.00		1.00		1.00	
Middle/High: 50,000 or more	0.79	(0.39–1.59)	0.73 *	(0.59–0.91)	0.74 *	(0.58–0.95)	0.51 *	(0.36–0.72)

* = *p* < 0.05.

## Data Availability

Restrictions apply to the availability of these data. Data were obtained from NAHDAP-ICPSR and are available at https://www.icpsr.umich.edu/web/NAHDAP/studies/36231/datadocumentation (accessed on 3 May 2022) with the permission of NAHDAP-ICPSR.
